# Brevetoxins and emergency department visits among children: A case-crossover study of Florida red tides

**DOI:** 10.1097/EE9.0000000000000481

**Published:** 2026-05-19

**Authors:** Erik M. Rizzo, Bianca Irimia Dowling, Ananya Ranganagoudar, Justin K. Arnold, Barbara Kirkpatrick, Catherine M. Bulka

**Affiliations:** a Department of Epidemiology, College of Public Health, University of South Florida, Tampa, Florida; b Judy Genshaft Honors College, University of South Florida, Tampa, Florida; c Department of Emergency Medicine, College of Medicine, University of South Florida, Tampa, Florida; d Florida Poison Information Center, Tampa, Florida; e Tampa General Hospital, Tampa, Florida; f Gulf of America Coastal Ocean Observing System (GCOOS), Department of Oceanography, Texas A&M University, College Station, Texas

**Keywords:** Harmful algal blooms, Children’s environmental health, Case-crossover

## Abstract

**Background:**

Algal blooms of *Karenia brevis* (“red tide”) produce brevetoxins that contaminate seawater, bioaccumulate in shellfish, and become aerosolized, exposing humans via ingestion and inhalation. While adult exposures are linked to respiratory and gastrointestinal symptoms, children—who may be especially vulnerable—remain understudied. We investigated whether short-term brevetoxin exposures trigger emergency department (ED) visits among children.

**Methods:**

We analyzed ED visits from coastal southwest Florida residents aged 0–18 years in the OneFlorida+ network (2012–2019) using a time-stratified case-crossover design with case and control days matched by week, day, month, and year. Two brevetoxin exposure indices were estimated for the prior 2 weeks (Lags_0–13_): (1) waterborne, based on spatially interpolated *K. brevis* concentrations linked to residential ZIP codes, and (2) aerosolized, incorporating wind speed and direction. Conditional logistic regression models estimated odds ratios per interquartile range increases in exposure indices, adjusting for time-varying covariates. Models were fit overall and by diagnosis group, including among a negative control group consisting of primarily trauma-related diagnoses.

**Results:**

Overall associations were null, as were associations for the negative controls. Stratified models showed: (1) same-day increases in waterborne and aerosolized exposures were associated with ~50% higher odds of respiratory visits (216 cases); (2) increases in waterborne exposures 12–13 days prior were modestly associated with ear, nose, throat, dental, and mouth diseases (321 cases); and (3) increases in aerosolized exposures 6–9 days prior were associated with ~50% higher odds of eye diseases (53 cases).

**Conclusions:**

Brevetoxins may trigger ED visits for respiratory, eye, and ear–nose–throat complaints among children in coastal southwest Florida. As red tides in the Gulf increase, public health guidance tailored to children may be needed.

**What this study adds**

This epidemiologic study is the first to specifically assess child health outcomes following red tides, harmful algal blooms that frequently occur along the southwest Florida coast. Unlike adults, children showed no increase in emergency department visits for gastrointestinal symptoms, likely due to infrequent shellfish consumption. However, exposures were associated with higher odds of emergency department visits for respiratory, eye, and ear, nose, and throat symptoms. Because these symptoms are common and nonspecific, clinicians should consider recent brevetoxin exposure in children presenting with such complaints, and families may benefit from tailored public health guidance to reduce exposure levels during blooms.

## Introduction

*Karenia brevis (K. brevis*), a dinoflagellate algae, frequently grows excessively or “blooms” in the Gulf waters of southwest Florida. These blooms occasionally discolor the water, giving it a reddish or brownish hue, and often produce noticeable odors due to the decay of dead marine life. However, the potent brevetoxins produced by *K. brevis* are tasteless, odorless, and invisible to the naked eye.^1^ These toxins pose health risks to humans through two primary exposure routes: (1) inhalation of aerosolized particles^2^ and (2) ingestion of contaminated bivalve shellfish^3^ or seawater.^4^ Brevetoxin exposure has been linked to respiratory irritation, gastrointestinal distress, and neurologic symptoms in adults.^3^ However, children—who breathe more air, eat more food, and drink more water relative to their smaller body sizes^5,6^—are underrepresented in the epidemiologic research. In addition to their higher per-body-weight potential intake of toxins, children have immature metabolic systems, including reduced capacity for hepatic detoxification, potentially increasing their susceptibility to environmental toxins.^5,7^

Although *K. brevis* blooms, colloquially referred to as “red tides,” have been recorded in the Gulf of America (formerly known as the Gulf of Mexico) since the 1800s,^1^ their frequency, duration, severity,^8^ and geographic range have increased in recent decades.^9–11^ Anthropogenic nutrient inputs, particularly nitrogen from agricultural runoff and septic systems,^8^ and ocean acidification and climate-related changes,^12,13^ are thought to contribute to bloom intensification and persistence. Some recent red tide events have lasted over a year, emphasizing the need for a clearer understanding of their public health implications.

*K. brevis* produces at least ten brevetoxin congeners (e.g., PbTx-1, PbTx-2),^14,15^ which activate voltage-sensitive sodium channels in cell membranes, causing prolonged neuronal depolarization and neuroexcitability.^16^ Ingestion of brevetoxins can occur through contaminated shellfish or (inadvertently) seawater.^3,4^ Although the Florida Department of Agriculture and Consumer Services enforces all commercial shellfish harvesting closures when *K. brevis* cell counts exceed 5,000 cells per liter of water,^17^ recreational harvesting during blooms remains a potential risk.^18^ Children tend to consume less shellfish than adults,^19^ but they are more likely to ingest seawater while swimming. Estimates suggest children aged 6–18 years swallow 68–71 ml per beach visit,^20^ contributing to overall exposure and potentially greater toxin burdens due to their small body mass.^5^

Inhalational exposure is another concern. The *K. brevis* organism is fragile and becomes lysed in the surf,^15^ releasing brevetoxins into the water. At the water’s surface, marine aerosols containing the brevetoxins form and can be blown inland by the wind. Even though published reports from the 1940s documented respiratory irritation following red tide events among coastal residents,^21^ little is known about how far aerosolized brevetoxins travel. Limited environmental surveillance has detected brevetoxins in air collected 6.4 km inland;^22^ however, extensive monitoring has yet to be performed at greater distances. High brevetoxin concentrations in the water, combined with strong and prevailing onshore winds and rough surf conditions, can promote wider dispersion,^14^ highlighting the need to assess health effects in populations living near bloom-prone coastlines. Children may be particularly vulnerable in this regard: although they have smaller absolute lung volumes than adults, they inhale a greater volume of air per kilogram of body weight due to higher ventilation rates,^23^ and therefore may receive disproportionately higher doses of aerosolized brevetoxins during *K. brevis* blooms.

Dermal absorption of brevetoxins remains an understudied route of human exposure. Brevetoxins are lipophilic and capable of penetrating biological membranes, with experimental evidence indicating they are absorbed through the epidermis, and accumulate preferentially within the dermal layers, which serve as a reservoir for continued slow release.^24^ Dermal contact is therefore thought to be more relevant for local irritant effects—including skin burning, itching, and rash—than for systemic toxicity. The high molecular weight of brevetoxins (~900 Da) may further limit skin penetration,^3,25^ though the extent of systemic absorption following dermal contact in humans remains incompletely characterized. Most human studies emphasize ingestion and aerosol exposure as primary concerns, with dermal contact considered a minor risk,^26^ although quantitative estimates of relative exposure magnitude and systemic bioavailability across inhalation, ingestion, and dermal routes remain unavailable for human populations, representing an important area for future research. Regardless of the precise contribution of each exposure route, and despite the potential for multiple exposure pathways to result in adverse health effects and children’s inherent physiologic susceptibility, no studies have specifically investigated the impacts of brevetoxin exposure in children. This study aimed to evaluate the hypothesis that short-term exposure to brevetoxins—both aerosolized and waterborne—is associated with increased emergency department (ED) visits among children residing in coastal southwest Florida.

## Methods

### Study population

We obtained ED visit electronic health records data from the OneFlorida+ Clinical Research Network.^27,28^ The network is one of the largest clinical data repositories in the state, including 14 Florida-based academic and health system partners, encompassing over 70 hospitals.^29^ The data follow the National Patient Centered Clinical Research Network Common Data Model,^30^ fostering harmonization across partner facilities and allowing for the capture of residential histories. As a HIPAA Limited Data Set, we were able to obtain actual dates of admission and patients’ residential ZIP codes (but not residential street addresses).

Because ZIP codes are not spatially defined areas, we used spatial proxies to facilitate the selection of the study area. For identifying eligible coastal areas, we matched patient residential ZIP codes to their corresponding 2020 ZIP Code Tabulation Areas (ZCTAs),^31^ which are geographic approximations developed by the U.S. Census Bureau. This allowed us to select ZIP codes located near the coastline by assessing ZCTA geometry. We used the 2020 Census ZCTA shapefile, which we downloaded from the U.S. Census Bureau’s Cartographic Boundary Files repository. We identified 64 ZIP codes with corresponding ZCTAs that were contiguous with or had a geometric center within 5 km of the Gulf of America and were located between latitudes 25.9 and 29.1°N, where sampling for *K. brevis* is routinely conducted.

ED visits by children (aged 0–18 years) between 1 January 2012 and 31 December 2019 were identified by the OneFlorida+ Data Trust based on residential addresses in one of the 64 coastal southwest Florida ZIP codes at the time of the visit (n = 4,353). We excluded visits where the residential ZIP code on the admission date could not be verified as “current” based on address history records (n = 1,520), and those where the verified ZIP code was more than 3 hours’ driving time from the facility’s ZIP code, suggesting the child may have been traveling and not at home immediately before visiting the ED (n = 247).

The OneFlorida+ Data Trust program operates under a University of Florida “data bank” Institutional Review Board protocol (IRB #: 201500466) and is structured as a HIPAA Limited Data Set, which requires a Data Use Agreement between the University of Florida and each contributing partner in place of individual patient consent or HIPAA authorization.^27^ The data provided to our research team was fully deidentified before transfer. On this basis, the University of South Florida’s Institutional Review Board determined that this study qualified for exempt status under federal regulations governing research involving existing deidentified data (USF IRB: Study007884).

### Study design

We used a time-stratified case-crossover design, in which each ED visit served as its own control.^32,33^ This design is well-suited for studying exposures that occur intermittently, have immediate and short-lived effects on risk, and are associated with abrupt outcomes.^34^ By comparing the risks of an ED visit over time, this design inherently controls for all time-stable child characteristics (e.g., sex, race/ethnicity) while the time-stratified approach accounts for seasonality and secular trends. We matched each case day (date of ED admission) to control days that fell on the same day of the week, within the same calendar month and year. This time-stratified matching structure controls for seasonality because case and control days share the same calendar month and year, ensuring that seasonal variation in both exposure and outcome affects case and control windows equally within each stratum; matching on year additionally controls for secular trends such as changes in ED utilization patterns or bloom frequency.^33^ To evaluate potential delayed effects of exposure, we also identified the 13 days preceding each case and control day, allowing for exposure windows of up to 2 weeks before each visit.

### *Karenia brevis* data source and assessment

We obtained *in-situ* measurements of *K. brevis* expressed as cells per liter, along with sampling dates and locations (latitude/longitude) from the National Oceanographic and Atmospheric Administration National Centers for Environmental Information’s Harmful Algal BloomS Observing System (HABSOS).^35^ HABSOS collates *K. brevis* monitoring data from several partners, including the Alabama Department of Environmental Management, Alabama Department of Public Health, U.S. Environmental Protection Agency, Florida Fish and Wildlife Research Institute, Integrated Ocean Observing System, Mississippi Department of Marine Resources, Texas Parks and Wildlife Department, University of South Florida, and U.S. Geological Survey. In total, 50,479 discrete measurements were collected from 19 December 2011 to 31 December 2019 between 83.5–81°W and 25–30°N. However, these data were patchy in both space and time (Supplemental Figure 1; https://links.lww.com/EE/A425), reflecting the event-driven nature of *K. brevis* monitoring efforts.^36^

To fill in the gaps, we performed the following workflow. First, we considered multiple samples collected at the same geographic coordinates on the same day to be replicates and calculated their average (n = 4,838) and replaced these duplicate values with the single averaged value. Then, we partitioned the HABSOS data by week. This analytic decision was supported by the fact that there were 456 days during the sampling period where no *K. brevis* measurements were available, and 2,264 days where fewer than 50 measurements were available. In contrast, when grouped by week, the average number of *K. brevis* measurements was 109 (range: 18–237). Next, we performed inverse distance weighting spatial interpolation for each week, using a projected coordinate system (UTM Zone 17N) to ensure accurate distance calculations. More details regarding the inverse distance weighting spatial interpolation are provided in the Supplemental Material; https://links.lww.com/EE/A425.

To link the predicted *K. brevis* concentrations to ED visits, we used population-weighted ZIP code centroids, rather than geometric centroids, as proxies for residential addresses.^37^ For each week, the nearest interpolated water point was identified and assigned, creating a weekly time series of estimated nearshore *K. brevis* concentrations for each coastal ZIP code. Two versions of the interpolated *K. brevis* dataset were used (see Supplemental Material; https://links.lww.com/EE/A425 for additional details): one that included bays, estuaries, and other sheltered waters, and another that excluded these areas, retaining only open coastal waters more directly exposed to wind. The version including sheltered waters was used to derive estimated waterborne brevetoxin concentrations, reflecting potential cumulative exposure through inhalation,^2^ ingestion,^3,4^ and, to a lesser extent, dermal absorption,^24^ while the version excluding sheltered waters was used to derive estimated aerosolized brevetoxin concentrations, reflecting potential exposure from inhalation only, as brevetoxin aerosolization is primarily driven by the mechanical action of wind-driven, breaking waves.^14,38^ *K. brevis* is known to consistently produce brevetoxins, and there is a well-established correlation between the concentration of *K. brevis* cells in the water and the amount of brevetoxins present.^39^ Therefore, water *K. brevis* concentrations serve as a reliable proxy for waterborne brevetoxins, which are not routinely monitored due to methodological constraints.^40^

### Meteorological data source and assessment

We obtained daily maps of modeled air temperature, relative humidity, and *u*- and *v*-wind components from the National Centers for Environmental Prediction North American Regional Reanalysis for the period 19 December 2011 to 31 December 2019.^41^ These data were provided on a ~32 km grid. Air temperature and relative humidity were predicted at 2 m above ground level and expressed in Kelvin and percent, respectively. The wind components were predicted at 10 m and represent the zonal (u) and meridional (v) vectors, expressed in meters/second. We calculated daily wind speed as √(u^2^ + v^2^) and wind direction using atan2(u, v), converting from radians to compass bearings by adding 180 ° and constraining values to 0–360 °. Daily air temperature and relative humidity values were extracted at ZIP code population-weighted centroids and used to calculate the daily heat index (a measure of perceived temperature) using the National Weather Service formula.^42^ Heat index values were converted to °C and structured as a daily time series at the ZIP code population-weighted centroid level.

To account for wind speed and direction, which mediate the relationship between brevetoxin levels in seawater and airborne concentrations over land,^38^ we applied an approach from the epidemiologic literature on pesticide exposures, intended to account for potential drift from application sites to residences.^43–46^ In brief, we drew 90 ° buffers anchored on the interpolated *K. brevis* point and extended to the nearest population-weighted ZIP code centroid. We then calculated the area-weighted average of daily wind directions and speeds within each wedge-shaped buffer. Wind direction values, originally representing the direction the wind was blowing from, were converted to wind vector directions (i.e., the direction the wind was blowing towards) by adding 180 ° and applying modulo 360. We then calculated the angular difference between each wind vector and the wedge-shaped buffer’s bearing and classified daily wind congruence as: onshore (0–45 °), crossshore (46–134 °), or offshore (135–180 °). A schematic illustrating how interpolated *K.* brevis cell counts served as the basis for both exposure indices (waterborne and aerosolized) is provided in Supplemental Figure 2; https://links.lww.com/EE/A425, while a diagram summarizing our workflow is provided in Supplemental Figure 3; https://links.lww.com/EE/A425.

### Air pollution data source and assessment

Aerosolized brevetoxins have a median diameter of 6–10 μm,^47^ small enough to be inhaled into human lungs. Their size makes them analogous to coarse particulate matter (e.g., PM_10_). To control for residual confounding by other types of air pollution, we obtained daily average PM_2.5_ (μg/m^3^) and daily maximum 8-hour ozone concentrations (ppb) for 19 December 2011 to 31 December 2019 from the U.S. Environmental Protection Agency’s Remote Sensing Information Gateway data repository.^48^ Specifically, we used the Fused Air Quality Surface Using Downscaling files, which integrate ground-level monitoring data with gridded output from the Community Multiscale Air Quality model. These fused datasets provide daily time series of air quality estimates at the geometric centroids of census tracts based on the 2010 U.S. Census. We performed nearest neighbor matching to assign the daily pollutant concentrations to the population-weighted ZIP code centroids.

### Outcome assessment

ED visits, including those admitted for inpatient stays, were classified according to the principal diagnosis code. These codes were based on either the International Classification of Diseases, Ninth Revision (ICD-9) or Tenth Revision (ICD-10) and could be for any cause (i.e., ICD-9: 001-V91 or ICD-10: A00-Z99). To group these diagnoses into a concise and clinically meaningful framework, we used the Pediatric Emergency Care Applied Research Network (PECARN) diagnosis grouping system, developed specifically for research on child ED visits.^49,50^ We then created a “negative control” group, consisting of diagnosis categories unlikely to be affected by brevetoxin exposures. The group included trauma; musculoskeletal and connective tissue diseases; child abuse; endocrine, metabolic, and nutritional diseases; genital and reproductive diseases; and neoplastic diseases (cancer, not benign neoplasms). The inclusion of negative outcome controls was aimed at detecting residual confounding and selection bias, thereby strengthening the internal validity of our study.^51^ Diagnosis categories considered for plausible associations with brevetoxin exposure included allergic, immunologic, and rheumatologic diseases; circulatory and cardiovascular diseases; diseases of the eye; ear, nose, throat (ENT), dental, and mouth diseases; fluid and electrolyte disorders; gastrointestinal diseases; hematologic diseases; neurologic diseases; psychiatric/behavioral diseases and substance abuse; respiratory diseases; skin, dermatologic, and soft tissue diseases; systemic states; toxicologic emergencies; and urinary tract diseases.

### Statistical analyses

We fit conditional logistic regression models, with ED visit serving as the stratum, to calculate odds ratios (ORs) and 95% confidence intervals (95% CIs) for the association between brevetoxin exposure and ED visits. Exposure-response and lag-response relationships were modeled using distributed lag nonlinear models.^52^ We explored linear parameterizations of exposures and lags and deviations from linearity by using natural splines with 3 ° of freedom. Model fits were compared using the Akaike information criterion (AIC). When the absolute ΔAIC exceeded 2,^53^ the model with the lower AIC was selected; otherwise, the spline model was retained by default to allow for flexibility in capturing nonlinear relationships.

Separate models were used to assess two distinct indices of brevetoxin exposure. The first exposure index was simply the assigned, interpolated *K. brevis* water concentration (cells/liter) from the nearest grid point in a given week, which we refer to as the waterborne brevetoxin index (WBI); this index was considered to represent nonaerosolized brevetoxin exposures potentially occurring via multiple routes. The second was an aerosolized brevetoxin index (ABI), calculated using the following equation:


$$ABI_{i,j}=KB_{w\left(i\right),\;p\left(\;j\right)}\times WS_{i,p\to j}\times WC_{i,p\to j}$$

where *i* refers to a given day, *w* refers to a given week, *j* refers to a given ZIP code (represented by the population-weighted centroid), *p* refers to the nearest interpolated water grid point within open coastal waters, KB refers to the interpolated *K. brevis* water concentration (cells/liter), WS refers to wind speed in a given wedge-shaped buffer (originally in meters/second, but converted to meters/day by multiplying by 86,400 seconds/day), and WC refers to the wind congruence in a given wedge-shaped buffer (onshore, crossshore, offshore). WC was arbitrarily weighted as 1 for onshore winds, 0.66 for crossshore winds, and 0.33 for offshore winds, which were hypothesized to confer the lowest exposure potential. The resulting ABI was expressed in (cells⋅meter)/(liter⋅day) and was considered to represent aerosolized brevetoxin exposures from inhalation only. For consistency across both indices, values at each lag were scaled by their respective interquartile ranges (IQR).

Models were adjusted for covariates measured on the day of the ED visit (Lag_0_), including average heat index, average PM_2.5_, and 8-hour maximum ozone, each modeled with natural splines and 3 ° of freedom, and a binary indicator for federal holidays. From these models, we extracted both single-day (Lag_0_, Lag_1_, …, Lag_13_) and cumulative (Lag_0–1_, Lag_0–2_,…,Lag_0–13_) measures of association. In addition to models of ED visits overall, we stratified models by diagnosis categories, including the negative control group. Categories with fewer than 50 cases were excluded to ensure statistical stability. Multiple sensitivity analyses were conducted to assess the robustness of the results; details are provided in the Supplemental Material; https://links.lww.com/EE/A425. All data processing, spatial analyses, and visualization were conducted in R version 4.4.2 (Vienna, Austria).

## Results

### Descriptive statistics

Overall, there were 2,586 ED visits by child residents of coastal southwest Florida between January 2012 and December 2019 (Supplemental Figure 4; https://links.lww.com/EE/A425). These cases were matched to 8,759 control days. The ratio of case to control days ranged from 1:1 to 1:4, with a median ratio of 1:2. The most common primary diagnosis group was trauma (376 cases), followed by ENT, dental and mouth diseases (321 cases), and gastrointestinal diseases (237 cases). Frequencies of the remaining diagnosis groups, as categorized using the PECARN system, are provided in Table 1. The cases had a mean age of 8 years (range: 0–18 years). Approximately half were female and half were male, and most were non-Hispanic white. Over half were covered by governmental health insurance. The majority lived fewer than 6 km from the Gulf; however, when considering only open coastal waters and excluding sheltered waters, nearly one-third resided 8 to 15 km away (Table 1). ED visits were most common during the first dry season (January to May), followed by the rainy season (June to September), and the second dry season (October to December).

**Table 1.** Patient and visit characteristics of the 2,586 ED visits in the OneFlorida+ Data Trust by child residents of coastal southwest Florida, 2012–2019

Characteristic	
Age (years), mean (SD)	8.2 (6.5)
Sex, N (%)	
Female	1,276 (49.3)
Male	1,310 (50.7)
Race/ethnicity, N (%)	
Non-Hispanic white	1,738 (67.2)
Non-Hispanic black	327 (12.6)
Hispanic	408 (15.8)
Non-Hispanic Asian or Pacific Islander	13 (0.5)
Another race/ethnicity	95 (3.7)
Missing	5 (0.2)
Insurance type, N (%)	
Governmental health insurance (including Medicaid)	1,490 (57.6)
Private insurance	393 (15.2)
No insurance	17 (0.7)
Other	425 (16.4)
Missing	261 (10.1)
Residential proximity to any Gulf waters^a^, N (%)	
<4 km	645 (24.9)
4–<6 km	952 (36.8)
6–<8 km	492 (19.0)
8–15 km	497 (19.2)
Residential proximity to open coastal Gulf waters^b^, N (%)	
<4 km	120 (4.6)
4–<6 km	993 (38.4)
6–<8 km	617 (23.9)
8–15 km	856 (33.1)
Season of ED visit, N (%)	
First dry season (January–May)	1,102 (42.6)
Rainy season (June–September)	828 (32.0)
Second dry season (October–December)	656 (25.4)
PECARN diagnosis group, N (%)	
Allergic, immunologic, and rheumatologic diseases	17 (0.7)
Child abuse^c^	5 (0.2)
Circulatory and cardiovascular diseases	25 (1.0)
Diseases of the eye	53 (2.0)
Endocrine, metabolic, and nutritional diseases^c^	85 (3.3)
ENT, dental, and mouth diseases	321 (12.4)
Fluid and electrolyte disorders	15 (0.6)
Gastrointestinal diseases	237 (9.2)
Genital and reproductive diseases^c^	49 (1.9)
Hematologic diseases	44 (1.7)
Musculoskeletal and connective tissue diseases^c^	176 (6.8)
Neoplastic diseases (cancer, not benign neoplasms)^c^	7 (0.3)
Neurologic diseases	131 (5.1)
Psychiatric/behavioral diseases and substance abuse	190 (7.3)
Respiratory diseases	216 (8.4)
Skin, dermatologic, and soft tissue diseases	142 (5.5)
Systemic states	204 (7.9)
Toxicologic emergencies (including environment)	6 (0.2)
Trauma^c^	376 (14.5)
Urinary tract diseases	41 (1.6)
Other/missing	246 (9.5)

a Distance from the nearest spatial interpolation grid point in any Gulf water to the ZIP code population-weighted centroid.

b Distance from the nearest spatial interpolation grid point in open Gulf water only (excluding sheltered water bodies such as bays, estuaries, and inlets) to the ZIP code population-weighted centroid.

c Collapsed into a negative control group (n = 698) with no expected plausible causal effect from brevetoxin exposures.

Distributions of exposure indices are presented in Table 2. The IQRs of the WBI were wider than those of the ABI, albeit in different units (cells/liter vs. [cells⋅meter]/[liter⋅day]).

**Table 2.** Distribution percentiles of waterborne and aerosolized brevetoxin exposure indices at each lag, overall and by case and control days

	All days (11,345 days)			Case days (2,586 days)			Control days (8,759 days)		
Time window	25th	50th	75th	25th	50th	75th	25th	50th	75th
Waterborne brevetoxin exposure index (cells/liter)									
Lag_0_	6	271	22,823	5	234	21,199	6	285	23,356
Lag_1_	6	271	22,037	5	244	21,199	6	285	22,216
Lag_2_	6	274	22,130	5	244	22,369	6	287	22,084
Lag_3_	6	262	22,130	5	233	21,264	6	280	22,245
Lag_4_	6	262	22,485	5	233	21,166	6	271	23,496
Lag_5_	6	260	22,485	5	262	20,931	6	259	23,702
Lag_6_	6	262	22,384	5	244	21,438	6	267	22,823
Lag_7_	6	271	23,080	5	260	22,737	6	274	23,356
Lag_8_	6	271	23,608	5	281	22,130	6	271	23,832
Lag_9_	6	270	23,966	5	283	23,580	6	262	23,966
Lag_10_	6	270	23,966	5	280	25,076	6	267	23,552
Lag_11_	5	267	23,966	5	283	25,145	6	261	23,485
Lag_12_	6	262	24,020	6	261	26,686	6	262	23,356
Lag_13_	6	262	24,020	6	255	26,686	6	262	23,608
Aerosolized brevetoxin exposure index ([cells⋅meter]/[liter⋅day])									
Lag_0_	8	411	39,138	7	361	40,696	8	423	38,789
Lag_1_	8	401	36,569	7	358	35,350	8	416	37,090
Lag_2_	8	404	39,414	7	318	38,896	8	436	39,499
Lag_3_	8	402	38,362	7	334	36,612	8	421	39,110
Lag_4_	8	408	39,593	7	376	40,018	8	420	39,582
Lag_5_	8	411	40,343	7	440	37,003	8	391	42,024
Lag_6_	8	399	38,142	7	385	33,918	8	401	39,814
Lag_7_	8	411	39,162	8	384	36,840	8	415	40,162
Lag_8_	8	399	39,064	8	376	34,815	8	407	40,033
Lag_9_	8	402	40,049	8	409	42,401	8	394	39,531
Lag_10_	8	407	40,527	8	394	41,755	8	415	40,118
Lag_11_	8	404	40,021	8	421	41,004	8	396	39,513
Lag_12_	8	403	41,287	9	386	44,838	8	409	40,193
Lag_13_	8	377	39,784	9	377	49,493	8	379	38,293

### Waterborne brevetoxins and emergency department visits

**Figure 1.**
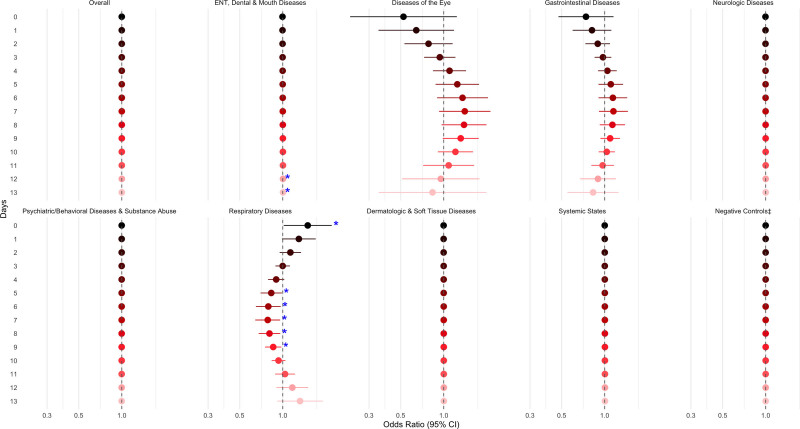
Adjusted† odds ratios for ED visits per an interquartile range increase in the waterborne brevetoxin index (cells/liter), overall and by diagnosis group, over single-day lags. †Adjusted for daily heat index (°C), ozone (ppb), PM_2.5_ (µg/m^3^), and federal holiday status (yes/no) on Lag_0_. ‡Negative controls include trauma; child abuse; musculoskeletal and connective tissue diseases; metabolic and nutritional diseases; genital and reproductive diseases; and neoplastic diseases. *P < 0.05.

**Figure 2.**
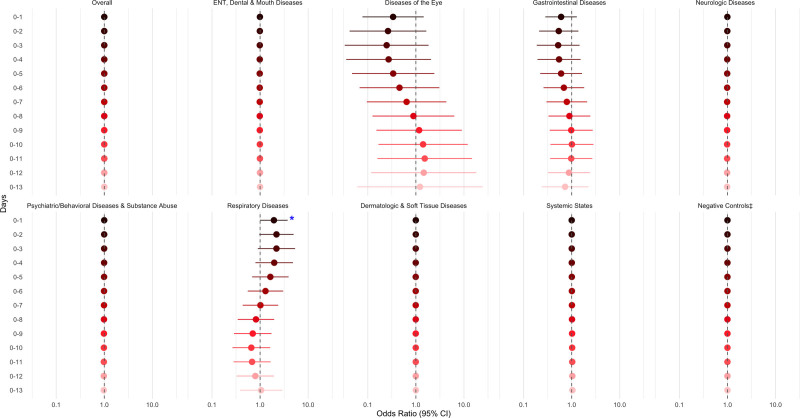
Adjusted† odds ratios for ED visits per an interquartile range increase in the waterborne brevetoxin index (cells/l), overall and by diagnosis group, over cumulative lags. †Adjusted for daily heat index (°C), ozone (ppb), PM_2.5_ (µg/m^3^), and federal holiday status (yes/no) on Lag_0_. ‡Negative controls include trauma; child abuse; musculoskeletal and connective tissue diseases; metabolic and nutritional diseases; genital and reproductive diseases; and neoplastic diseases. *P < 0.05.

Associations between the WBI and overall ED visits were null across both single-day (Figure 1) and cumulative lags (Figure 2). These results are based on a linear parameterization of the index, implying that the null effects are assumed constant across the exposure range. Similar null associations were observed for the negative control outcomes; diseases of the eye; gastrointestinal diseases; neurologic diseases; psychiatric/behavior diseases, substance abuse; skin, dermatologic, and soft tissue diseases; and systemic states.

In contrast, higher waterborne brevetoxin levels at 12- and 13-day lags (Lag_12_, Lag_13_) were significantly associated with increased odds of ED visits for ENT, dental, and mouth diseases, although the effect sizes were modest (Figure 1; Supplemental Table 1; https://links.lww.com/EE/A426).

For respiratory diseases, a more nuanced pattern emerged: same-day exposure (Lag_0_) was associated with approximately 50% higher odds of an ED visit (95% CI = 2%, 120%), while exposures 5 to 9 days prior (Lag_5_, Lag_6_, Lag_7_, Lag_8_, Lag_9_) were associated with 14%–22% lower odds (95% CIs = 2%, 36%). These associations were estimated using a model allowing for a nonlinear relationship between waterborne brevetoxin levels and respiratory-related ED visits (Figure 1; Supplemental Table 1; https://links.lww.com/EE/A426). A 1-IQR increase in average exposure on the day of and the day before the ED visit was associated with nearly double the odds of a respiratory ED visit (OR = 1.94, 95% CI = 1.00, 3.74; Figure 2, Supplemental Table 1; https://links.lww.com/EE/A426).

Sensitivity analyses restricting the dataset to exclude closely spaced repeat visits (<90 days apart) attenuated some previously significant associations; however, the magnitude and direction of the estimates for respiratory-related ED visits remained largely unchanged (Supplemental Table 2; https://links.lww.com/EE/A426). Stratified analyses suggested slightly lower risks among individuals living closer to the water for respiratory, ENT, dental, and mouth-related visits (Supplemental Figure 5; https://links.lww.com/EE/A425), and slightly lower risks during the dry season for respiratory-related visits (Supplemental Figure 6; https://links.lww.com/EE/A425).

### Aerosolized brevetoxins and emergency department visits

**Figure 3.**
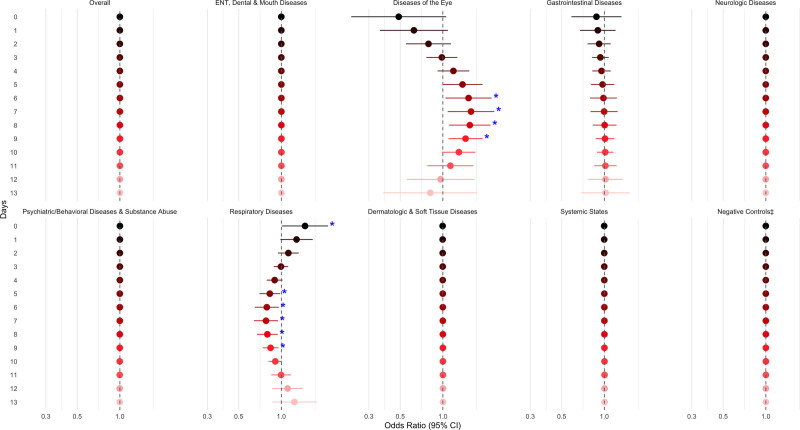
Adjusted† odds ratios for ED visits per an interquartile range increase in the aerosolized brevetoxin index ([cells⋅meter]/[liter⋅day]), overall and by diagnosis group, over single-day lags. †Adjusted for daily heat index (°C), ozone (ppb), PM_2.5_ (µg/m^3^), and federal holiday status (yes/no) on Lag_0_. ‡Negative controls include trauma; child abuse; musculoskeletal and connective tissue diseases; metabolic and nutritional diseases; genital and reproductive diseases; and neoplastic diseases. *P < 0.05.

**Figure 4.**
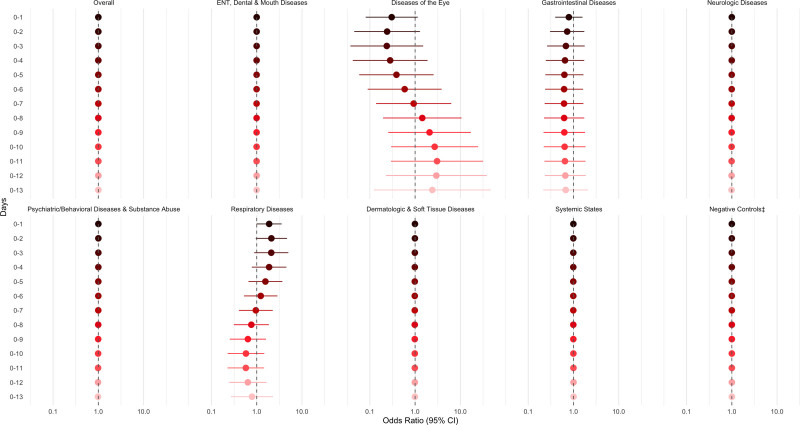
Adjusted† odds ratios for ED visits per an interquartile range increase in the aerosolized brevetoxin index ([cells⋅meter]/[liter⋅day]), overall and by diagnosis group, over cumulative lags. †Adjusted for daily heat index (°C), ozone (ppb), PM_2.5_ (µg/m^3^), and federal holiday status (yes/no) on Lag_0_. ‡Negative controls include trauma; child abuse; musculoskeletal and connective tissue diseases; metabolic and nutritional diseases; genital and reproductive diseases; and neoplastic diseases. *P < 0.05.

Associations between the ABI and overall ED visits were null across both single-day (Figure 3) and cumulative lags (Figure 4). Null associations were also observed for the negative control outcomes; ENT, dental, and mouth diseases; gastrointestinal diseases; neurologic diseases; psychiatric/behavior diseases, substance abuse; skin, dermatologic, and soft tissue diseases; and systemic states.

However, results from the spline model indicated that same-day exposure to higher aerosolized brevetoxin levels (Lag_0_) was associated with 47% (95% CI = 1%, 114%) higher odds of an ED visit for respiratory diseases. Conversely, higher exposures on days 5 to 9 prior (Lag_5_, Lag_6_, Lag_7_, Lag_8_, Lag_9_) were again associated with lower odds of ED visits for respiratory diseases (Figure 3; Supplemental Table 3; https://links.lww.com/EE/A426). These associations remained robust after excluding repeat visits occurring <90 days apart (Supplemental Table 4; https://links.lww.com/EE/A426). Stratified models by residential proximity to water showed overlapping confidence intervals (Supplemental Figure 7A; https://links.lww.com/EE/A425), suggesting no significant effect modification by distance. However, seasonal stratification indicated that the dry season may offer greater protection against respiratory ED visits than the rainy season (Supplemental Figure 7B; https://links.lww.com/EE/A425).

A positive association was also observed between aerosolized brevetoxin exposure and diseases of the eye. Specifically, 1-IQR increases in exposures 6 to 9 days before an ED visit (Lag_6_, Lag_7_, Lag_8_, Lag_9_) were associated with 45%–58% higher odds of eye-related ED visits (95% CIs = 4%, 130%) in spline models (Figure 3; Supplemental Table 3; https://links.lww.com/EE/A426). Due to the small number of cases (n = 53), subgroup analyses to assess differences in susceptibility and sensitivity analyses to exclude closely spaced repeat visits were not feasible. Finally, although we planned sensitivity analyses to further control for the influence of tropical cyclones, these events were rare during the study period (Supplemental Table 5; https://links.lww.com/EE/A426) and therefore would not have meaningfully affected the estimated associations between brevetoxin exposures and ED visits.

## Discussion

Red tide events in the Gulf of America occurred repeatedly during the study period, with major blooms documented in 2012, 2015–2016, and 2017–2018.^36^ The results of this time-stratified case-crossover study suggest that exposures to brevetoxins during these events may contribute to acute health issues among children living in coastal areas. Same-day exposure to aerosolized brevetoxins was associated with 50% higher odds of respiratory-related ED visits. Positive associations were also observed between aerosolized brevetoxins and eye diseases, and between waterborne brevetoxins and ENT, dental, and mouth diseases, though at longer lag periods. The positive association observed for respiratory diseases is consistent in direction with findings from existing studies of predominantly adult populations, which have reported increased emergency and nonemergency visits for diagnoses such as asthma, bronchitis, pneumonia, and upper airway diseases.^54–56^ A direct comparison of effect magnitudes across studies, however, is limited by differences in exposure assessment and study design, as prior work has relied on binary bloom versus nonbloom period comparisons,^54^ or waterborne *K. brevis* cell counts assigned at the county- or ZIP code-level,^55,56^ rather than estimates of aerosolized brevetoxin concentrations derived from integrated satellite imagery, in situ measurements, and meteorological data. Nevertheless, these studies provide consistent epidemiologic evidence of respiratory health effects associated *with K.* brevis blooms in general populations. While prior studies have also reported increased gastrointestinal ED admissions during active red tide blooms,^55,57^ we did not observe similar associations among children, which may reflect differences in exposure pathways or age-related behavioral patterns. To our knowledge, this is among the first studies to report brevetoxin-associated increases in ED visits for eye diseases and ENT, dental, and mouth conditions in children, extending the existing literature on harmful algal bloom health impacts to a population that has received little prior attention.

The observed immediate effects of brevetoxin exposure on respiratory health also align with the prior descriptive accounts and established biological mechanisms of these toxins. When inhaled, brevetoxins can cause bronchoconstriction and airway inflammation.^58^ Previous studies have documented acute respiratory symptoms during red tide events, including coughing, wheezing, and throat irritation, particularly among beachgoers and coastal residents.^54,59^ However, the protective associations observed at 5–9 day lags warrant careful interpretation and may have multiple explanations. First, children experiencing acute respiratory symptoms during peak exposure periods may modify their behavior, for example, by temporarily avoiding outdoor activities, thereby reducing subsequent exposure risk. This “harvesting” effect, where susceptible individuals experience health events earlier during high-exposure periods,^60^ could manifest as apparent protective effects in the days following brevetoxin exposure as the pool of susceptible children becomes depleted. Second, children who visited the ED during high-exposure periods may have been prescribed medications (e.g., bronchodilators, corticosteroids) that provided continued symptom relief, reducing the likelihood of repeat visits in subsequent days. Our sensitivity analyses excluding closely spaced repeat visits did not substantially alter these patterns, suggesting that the delayed protective associations are not solely attributable to within-individual correlation of exposures and outcomes.

The positive association between aerosolized brevetoxins and eye diseases at lag days 6 to 9 is biologically plausible, given that a burning sensation in the eyes is frequently reported.^3,59^ The delayed timing of these associations may reflect the natural history of eye conditions, where initial mild irritation progresses to more severe symptoms requiring medical attention several days postexposure.^61^ However, the small number of eye-related cases (n = 53) limits our ability to draw firm conclusions, and these findings require replication in larger studies. Similarly, the modest associations between waterborne brevetoxins and ENT, dental, and mouth diseases at lag days 12 and 13 suggest potential delayed effects on oral and upper respiratory tissues. While less commonly reported than respiratory or eye symptoms, oral mucosa irritation has been documented following consumption of contaminated seafood.^62^ The extended lag period may indicate progression of initially mild symptoms to conditions requiring emergency care. However, the heterogeneity of this diagnosis group,^49,50^ which encompasses conditions ranging from dental trauma to pharyngitis, complicates mechanistic interpretation.

Our study employed novel spatiotemporal exposure indices that integrated water sampling data with environmental factors influencing brevetoxin transport and aerosolization. The waterborne index directly incorporated measured *K. brevis* cell counts, while the aerosolized index additionally accounted for wind speed and direction, which are critical determinants of onshore brevetoxin transport.^22,38^ This approach represents an advancement over studies relying solely on cell counts or proximity to monitoring stations, though residual exposure misclassification remains inevitable given the spatial heterogeneity of red tide blooms and individual variation in time-activity patterns. Interestingly, our stratified analyses by residential distance to water suggested slightly lower risks for respiratory outcomes associated with waterborne brevetoxin exposures among children living closer to the coast. One speculative explanation is that coastal residents may develop tolerance to brevetoxins through more frequent low-level environmental exposure, resulting in attenuated acute responses — a pattern consistent with findings from Kirkpatrick et al.,^63^ though the underlying biological mechanism has not yet been directly tested.

Several limitations of our study warrant consideration. First, the *K. brevis* data underlying our exposure assessment approach were aggregated by week, whereas the wind data were available at a daily resolution. The resulting exposure indices were therefore assumed to reflect daily conditions, and lag-specific findings should be interpreted with this temporal imprecision in mind. Second, our exposure assessment relied on residential ZIP codes and lacked individual-level behavioral or activity data, which may not capture personal exposure levels (particularly for children whose time-activity patterns vary substantially), thus precluding strong causal inference. Third, ED visits represent the more severe end of the morbidity spectrum; we could not assess milder symptoms managed at home or in primary or urgent care settings, and ED utilization patterns may vary seasonally or differ among children with varying access to healthcare. Fourth, the small number of cases for certain outcome categories (e.g., eye diseases) limited our ability to conduct comprehensive subgroup analyses. Fifth, we were unable to assess effect modification by preexisting disease status, such as asthma. Given the high prevalence of asthma in children and the respiratory mode of action of brevetoxins, future studies should specifically investigate exposure-outcome relationships in this susceptible subpopulation. Sixth, the negative control outcome group was composed of heterogeneous diagnosis categories, which may differ in healthcare-seeking patterns and relationships with socioeconomic determinants of healthcare access. To the extent that coastal residence — and thus brevetoxin exposure — is associated with socioeconomic characteristics that influence healthcare utilization, this heterogeneity could introduce differential bias across negative control conditions, potentially obscuring or mimicking evidence of residual confounding. Additionally, although the time-stratified case-crossover design inherently controls for time-invariant individual characteristics, it does not fully account for short-term time-varying confounders. While we controlled for ozone and particulate matter, we were unable to adjust for other time-varying exposures, such as pollen that may cooccur with bloom periods. That said, the limited temporal overlap between peak *K. brevis* bloom activity and peak respiratory virus circulation in this region reduces the likelihood of substantial confounding by seasonal viral illnesses. Finally, our study population was limited to coastal southwest Florida, where red tide events caused by *K. brevis* occur regularly and can persist for extended periods. Generalizability to other geographic regions experiencing harmful algal blooms caused by different organisms warrants caution.

Still, this study benefits from several strengths, including a case-crossover design that inherently controls for time-invariant individual characteristics; incorporation of time-varying factors such as heat index and other air pollutants; a large sample of ED visits spanning more than 8 years; comprehensive diagnosis categorization capturing multiple outcomes, based on the PECARN system; and spatially-refined exposure assessment accounting for environmental transport factors. The time-stratified matching approach eliminates concerns about temporal confounding from seasonal trends and long-term changes in healthcare utilization patterns. As the first epidemiologic study of Florida red tides that is specific to children, our findings provide critical insights into a vulnerable population.

Our study provides preliminary evidence that brevetoxin exposures during Florida red tide events contribute to an increase in pediatric ED visits in coastal southwest Florida. If confirmed by future research, the results would underscore the need for targeted risk communication and enhanced clinical awareness for families with children.^64^ For instance, public health guidance could direct caregivers to resources like the Gulf Coast Red Tide Respiratory Forecast,^65^ which uses *K. brevis* water concentrations and wind data to predict respiratory irritation risk at individual beaches up to 36 hours in advance. These forecasts can help families make informed decisions—such as limiting outdoor activities—during peak bloom periods. In clinical settings, improving awareness of harmful algal blooms may also be important.^66^ Because brevetoxin-related symptoms are common and nonspecific, asking patients about recent environmental exposures or checking local red tide advisories could help clinicians distinguish toxin-related illness from other causes, thereby avoiding unnecessary treatments such as antibiotics for a presumed infection.

In conclusion, this study suggests that short-term brevetoxin exposure from the Florida red tide is associated with acute respiratory morbidity in children. Additional associations were observed for eye diseases and ENT conditions, suggesting multisystem health impacts. As climate change and nutrient pollution are projected to increase the frequency, duration, and geographic range of harmful algal blooms,^8,12,13^ understanding and mitigating health impacts on vulnerable populations such as children will become increasingly critical.

## Conflicts of interest statement

The authors declare that they have no conflicts of interest with regard to the content of this report.

## ACKNOWLEDGMENTS

*Research reported in this publication was supported in part by the OneFlorida+ Clinical Research Network, funded by the Patient-Centered Outcomes Research Institute numbers CDRN-1501-26692, RI-CRN-2020-005, and RI-FLORIDA-01-PS1; in part by the University of Florida Clinical and Translational Science Institute, which is supported in part by the NIH National Center for Advancing Translational Sciences under award numbers UL1TR001427 and UL1TR000064. The content is solely the responsibility of the authors and does not necessarily represent the official views of the Patient-Centered Outcomes Research Institute (PCORI), its Board of Governors or Methodology, the OneFlorida+ Clinical Research Network, the UF-FSU Clinical and Translational Science Institute, or the National Institutes of Health.*

## Supplementary Material
