# Supplementary material for: Brevetoxins and emergency department visits among children: A case-crossover study of Florida red tides
Source: Environ Epidemiol. 2026 May 19;10(3):e481. doi: 10.1097/EE9.0000000000000481 (PMC13189565; doi:10.1097/EE9.0000000000000481)
Supplement: Supplementary file 1 [file ee9-10-e481-s001.pdf]

SUPPLEMENTAL MATERIAL

Spatial Interpolation of Weekly K. brevis Concentrations in Gulf Waters

To perform spatial interpolation of weekly *K. brevis* concentrations, we generated a 100 x 100 point grid spanning the longitudinal and latitudinal range of the data. Grid points falling on land, defined using a detailed shapefile of the Gulf shoreline,[67] were masked. The IDW power parameter (*idp*), which controls the influence of distant points, was optimized to minimize interpolation error. A stratified random sample of 15% of weeks, representing the full range of bloom intensities (from background, defined as a maximum *K. brevis* concentration of ≤1,000 cells/Liter, to high, defined as a maximum of >1,000,000 cells/Liter), was used for this calibration. For each sampled week, IDW was performed across a range of *idp* values from 0.5 to 3.5 in increments of 0.1 to find the most accurate model. Model performance for each *idp* value was evaluated using 10-fold cross-validation, with Root Mean Square Prediction Error (RMSPE) serving as the accuracy metric. An *idp* value of 1.3 was selected, as it consistently yielded the lowest mean RMSPE across the diverse bloom conditions in the test set. Using the optimized *idp* of 1.3, IDW interpolation was performed for each week in the study period, resulting in weekly interpolated surfaces of predicted *K. brevis* concentrations at every grid point in the study area.

The weekly interpolated surfaces were then subjected to a manual filtering process to remove any interpolated points falling within bays, estuaries, and other sheltered waters; this ensured that predicted *K. brevis* concentrations to be used in analyses of aerosolized brevetoxins were limited to open coastal waters, consistent with prior observations that the wave and wind conditions necessary for aerosolization and inland transport are substantially diminished in sheltered waters.[22,38,68,69]

Sensitivity Analyses

Tropical cyclones have a complex relationship with algal blooms. By physically disrupting the water column with strong winds, tropical cyclones can temporarily dissipate blooms.[70] However, nutrient runoff from land following tropical cyclones can also stimulate new or worsen existing blooms weeks later.[71,72] In addition, tropical cyclones can cause injuries, infectious diseases, stress-induced conditions, and discontinuity of healthcare that result in increased ED visits afterward,[73-76] despite lower ED visit rates *during* storms due to evacuations or hazardous conditions.[77] To link ED visits to tropical cyclone data, we used the *hurricaneexposure* (Version 0.1.1) and *hurricaneexposuredata* (Version 0.1.0) R packages to obtain data on all tropical cyclones that occurred between 2012 and 2019.[78,79] We calculated the minimum distance between each hurricane's track and county population centers using the Haversine formula and restricted our exposure definition to tropical cyclones passing within 100 kilometers of county centroids. ZIP codes were mapped to counties using quarterly ZIP-to-census tract crosswalk files from the U.S. Department of Housing and Urban Development.[80] The Saffir-Simpson Scale was used to categorize tropical cyclones by maximum 1-minute sustained surface winds measured 10 meters above the ground. We retained tropical storms and hurricanes of categories 1 or higher, then linked each to residential ZIP codes by date. Upon implementing this linkage, we found that only 3 case days and 17 control days in our study population were exposed to a same-day tropical cyclone (**Supplemental Table 5**). Given this extremely small number of exposed days, we determined that quantitatively adjusting for same-day tropical cyclone exposure in our conditional logistic regression models was not practical, as the near-identical overlap between the primary models and those that would include this covariate would not be materially different.

To explore potential effect modifiers, we fitted stratified models for exposure-outcome associations that were identified as statistically significant (i.e., where any coefficient for a brevetoxin exposure index had $p < 0.05$). Stratification was based on residential proximity to the water and the season of the ED visit. Residential proximity was classified as <6 or ≥6 km between the population-weighted centroid of the residential ZIP code and the nearest interpolated *K. brevis* point, using the grid with sheltered waters for waterborne brevetoxin exposures and the grid without for aerosolized brevetoxin exposures. Season was classified as dry (January-May or October-December) or rainy (June-September).

Some patients had multiple ED visits during the study period. To preserve clinically distinct episodes while minimizing the potential for correlation between closely spaced repeat visits, we limited the dataset to one ED visit per patient, unless repeat visits were separated by more than 90 days. This process resulted in the exclusion of 761 visits that occurred ≤90 days after a previously retained visit. Conditional logistic regression models were then re-run on this smaller subset to assess the robustness of the results to the assumption of independent observations.

SUPPLEMENTAL FIGURES

Supplemental Figure 1. Hovmöller diagram showing the frequency of *in-situ K. brevis* measurements available in the Harmful Algal BloomS Observing System (HABSOS) for the study area and time period, by week and latitude

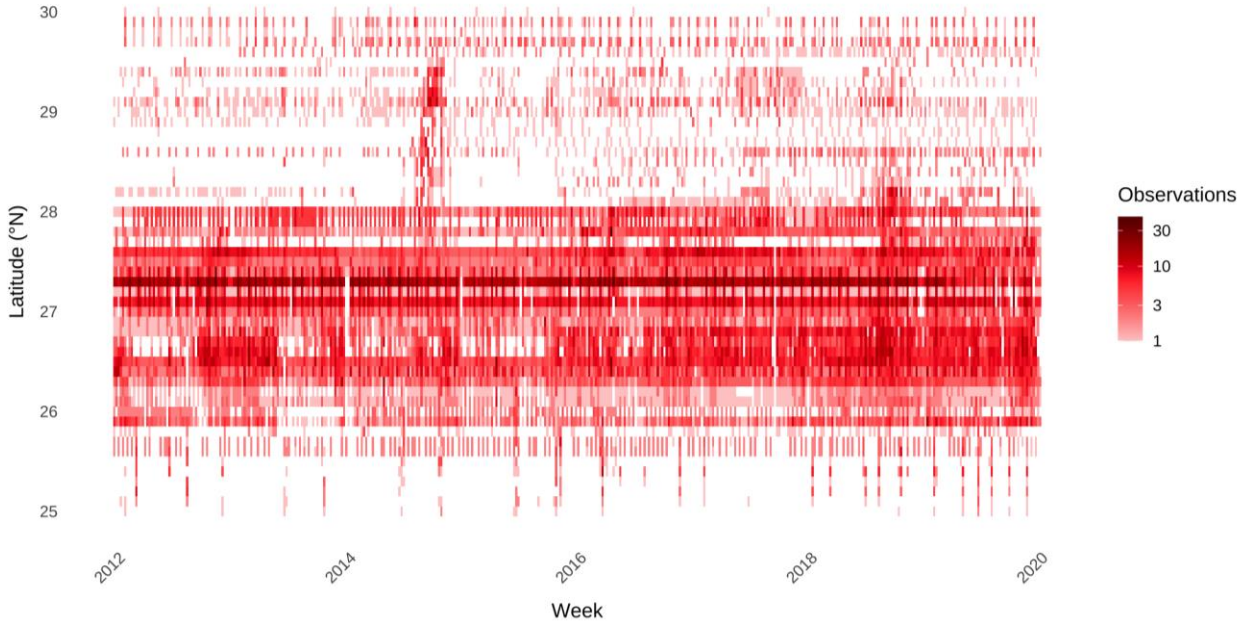

Supplemental Figure 2. Schematic describing our spatiotemporal exposure assessment framework for constructing brevetoxin indices

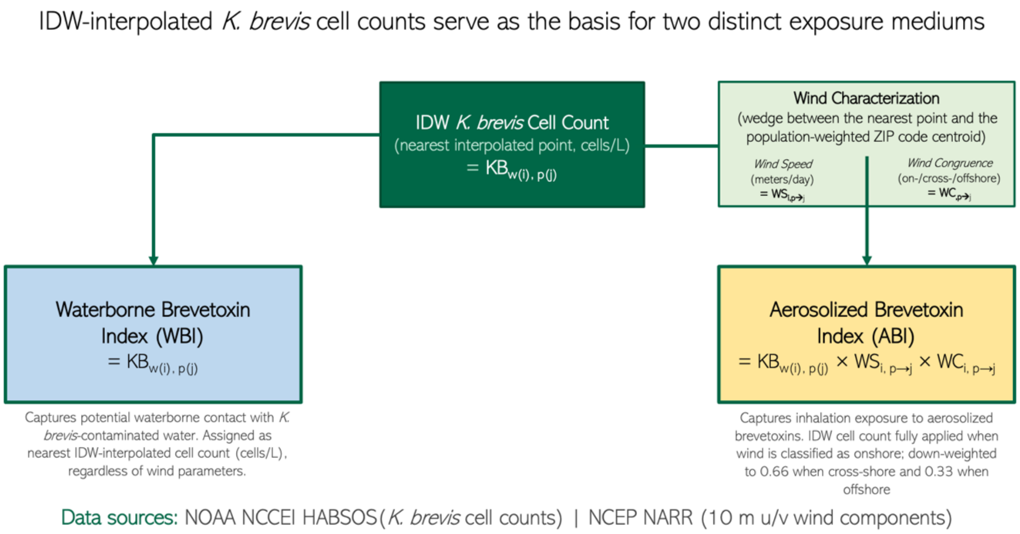

Supplemental Figure 3. Workflow for analyzing *K. brevis* and wind data to assess aerosolized exposure levels

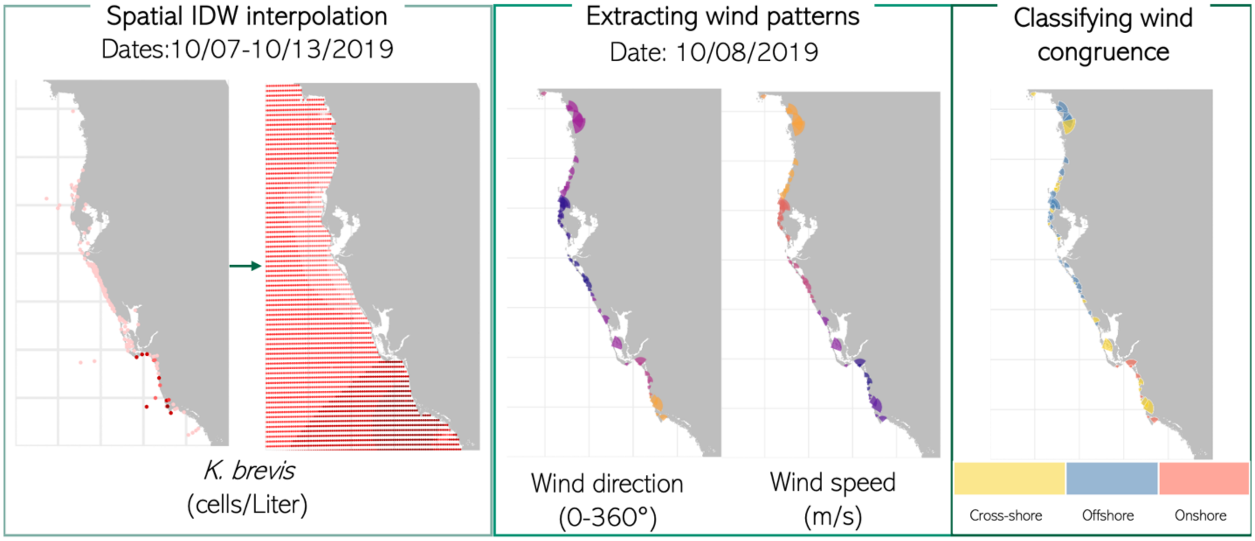

Supplemental Figure 4. Flow diagram of inclusion and exclusion criteria for ED visits by children residing in southwest coastal FL, OneFlorida+ Data Trust 2012-2019

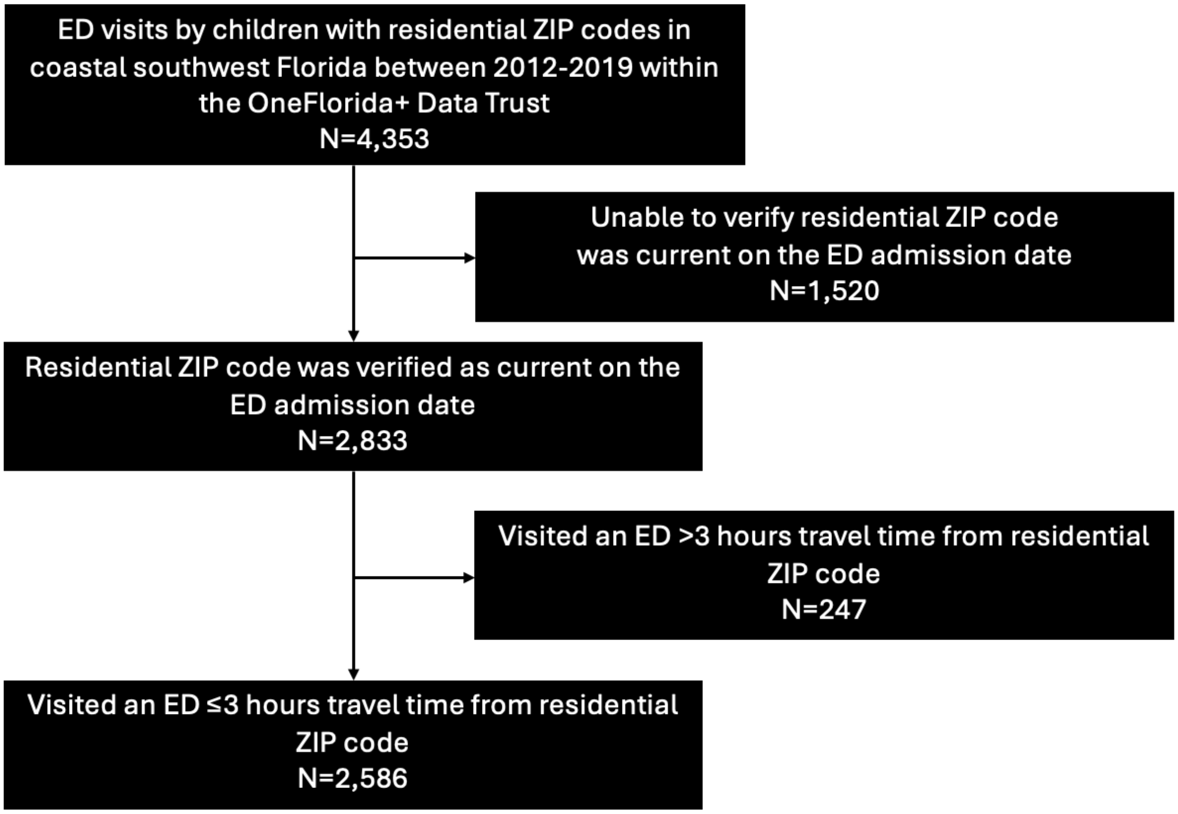

Supplemental Figure 5. Adjusted† odds ratios for ED visits per an interquartile range increase in the waterborne brevetoxin index (cells/Liter) over single-day lags, stratified by residential proximity to any Gulf waters, for respiratory diseases (A) and ear, nose, throat (ENT), dental & mouth diseases (B)

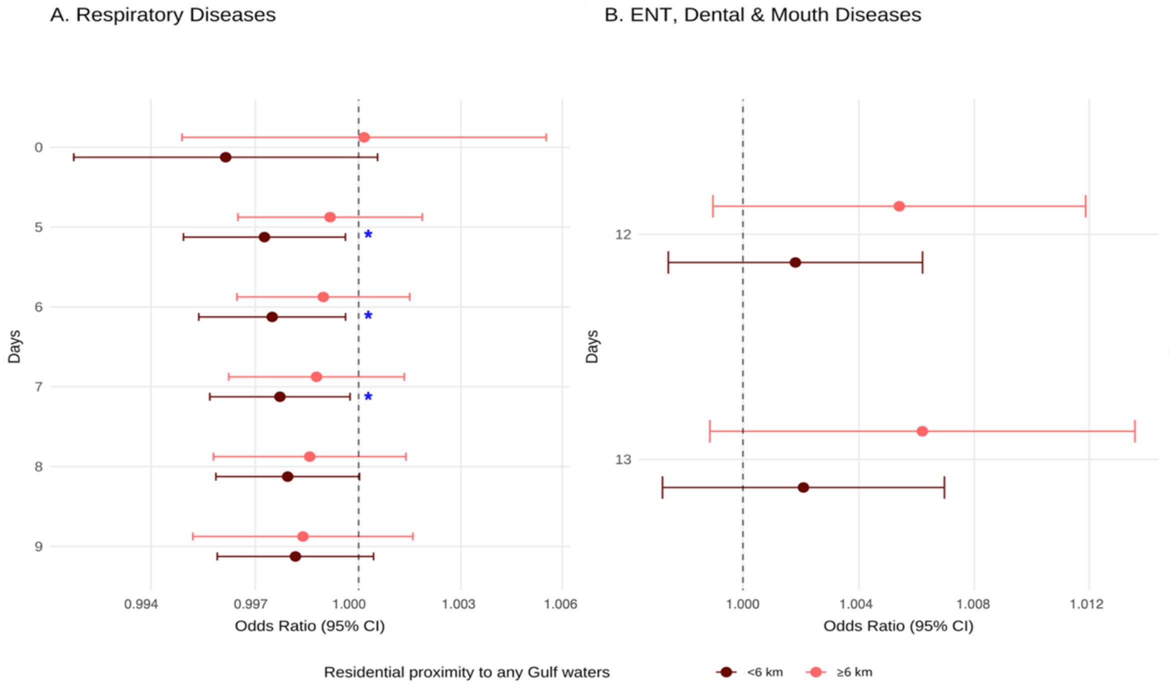

† Adjusted for daily heat index (°C), ozone (ppb), $PM_{2.5}$ (µg/m^3), and federal holiday status (yes/no) on Lag_0

* $p < 0.05$

Supplemental Figure 6. Adjusted† odds ratios for ED visits per an interquartile range increase in the waterborne brevetoxin index (cells/Liter) over single-day lags, stratified by season, for respiratory diseases (A) and ear, nose, throat (ENT), dental & mouth diseases (B)

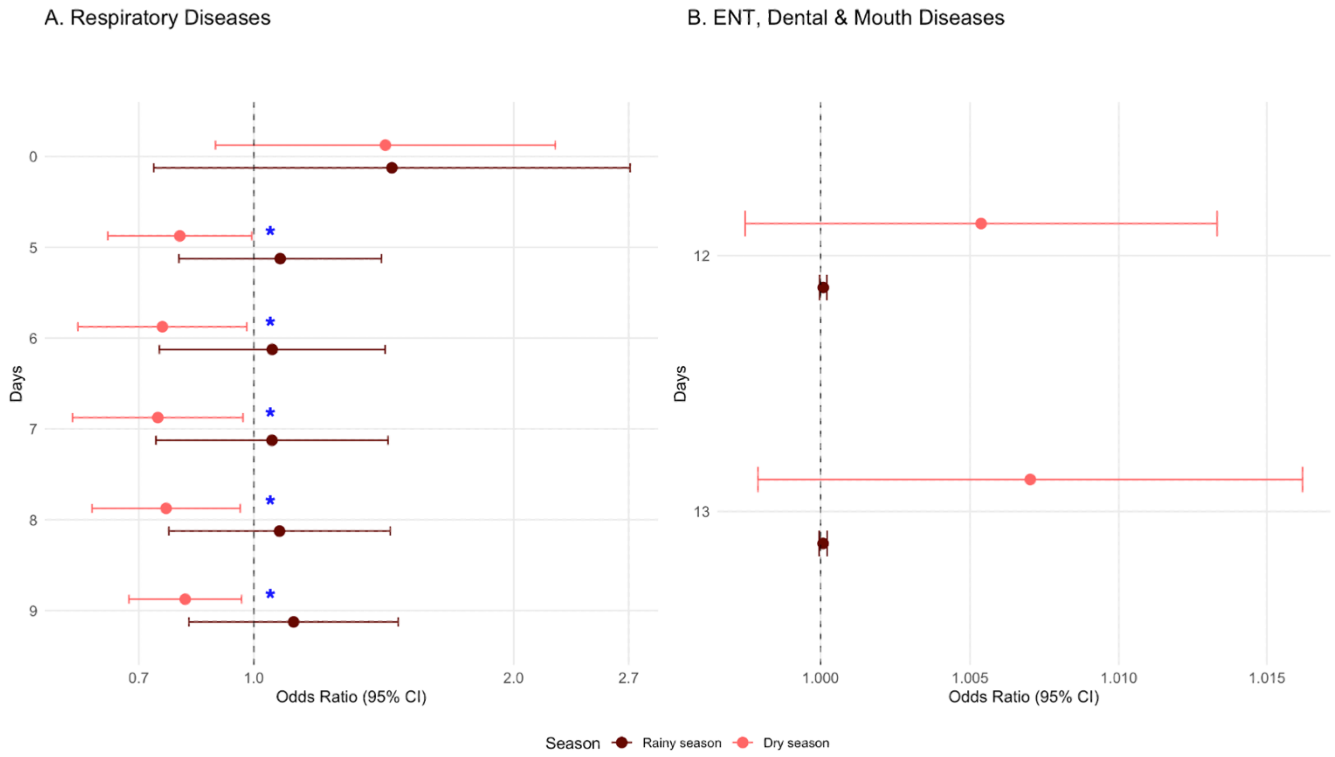

† Adjusted for daily heat index (°C), ozone (ppb), $PM_{2.5}$ (µg/m^3), and federal holiday status (yes/no) on Lag_0
* $p < 0.05$

Supplemental Figure 7. Adjusted† odds ratios for ED visits for respiratory diseases per an interquartile range increase in the aerosolized brevetoxin index ([cells·meter]/[Liter·day]) over single-day lags 0 through 9, stratified by residential proximity to open coastal Gulf waters (A) and season (B)

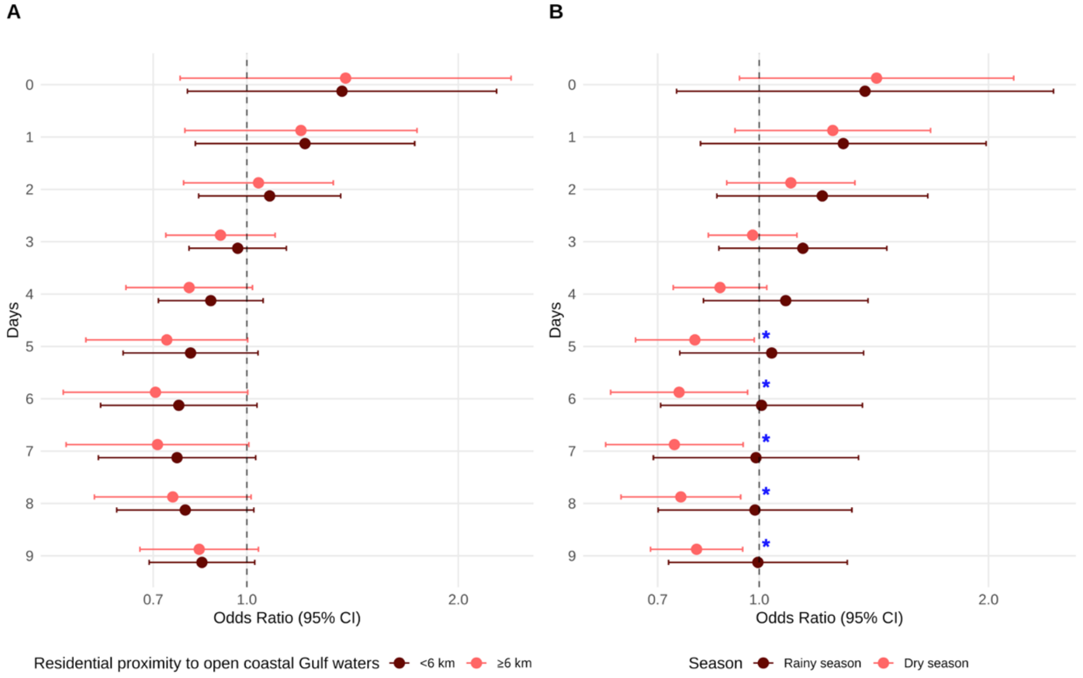

† Adjusted for daily heat index (°C), ozone (ppb), $PM_{2.5}$ ($\mu g/m^3$), and federal holiday status (yes/no) on Lag_0

* $p < 0.05$
